# Alcohol and Infancy

**Published:** 1920-02-21

**Authors:** 


### Alcohol and Infancy.

In view of the figures and results now appearing after relaxation of the war-time liquor control, and the sundry benefits which we think can be attributed to the measures adopted in 1915, which have probably saved the lives of many infants, the address given by Dr. C. W. Saleeby, the Chairman of the National Birth Rate Commission, may well be quoted. Recently it has been issued in pamphlet form, and urges the importance, in the interests of the race, that some effective control should be permanently maintained. It is not a question of prohibition, and the whole forms a very logical statement to show the relation between alcohol and infancy.

Very briefly we may summarise a few of the outstanding points. The biological action, now relatively well established, is mainly that alcohol intoxicates or poisons the germ plasm—*i.e.*, the future, in the person of the future parent, and this in both sexes. The work of Stockard experimentally demonstrated this action, and many instances of ascertained family history complete the evidence required.

Then there is the influence of alcohol in the child during gestation, or indirectly *viâ* the milk. The distribution of alcohol in the body of the prospective mother is well known, but a point is made against the popular superstition among the poor that stout and porter are good for motherhood. The vitamine question arises. A mother must obtain these substances in her food; but, though they are abundantly found in barley, malt, and yeast, yet no trace of them is left in beer.

Overlying is a notorious cause of infant mortality; and, though the connection of alcohol with this happening is not strictly biological, yet the influence of alcoholic sleep and drunkenness is obvious. During the control, the pronounced predominance of "Saturday night" for these deaths had largely disappeared, and it requires no stretch of the imagination to propose that the total saving of life was a feature not to be lightly discarded. Another point is that the babies of an alcoholic mother are probably more easily suffocated than are the normal.

Of seasonal and environmental influences, some note is taken; but more important is the question of the bearing on venereal disease, which in question of infant mortality is now almost pre-eminent. According to Dr. Saleeby, the relations of alcohol to venereal disease are fivefold. Let us record them after him. Alcohol lowers the resistance to temptation, alcohol lowers the resistance to infection, alcohol interferes with disinfection, alcohol aggravates the symptoms, and alcohol prejudices the treatment.

In these ways, by helping venereal disease, alcohol undoubtedly increases the mortality figures, apart from its more direct effects.

